# An Immune-Gene-Based Classifier Predicts Prognosis in Patients With Cervical Squamous Cell Carcinoma

**DOI:** 10.3389/fmolb.2021.679474

**Published:** 2021-07-05

**Authors:** Huixia Yang, Xiaoyan Han, Zengping Hao

**Affiliations:** Department of Gynecology and Obstetrics, Beijing Friendship Hospital, Capital Medical University, Beijing, China

**Keywords:** cervical squamous cell carcinoma, immune, gene, PD1/PD-L1, prognostic, nomogram, signature

## Abstract

**Objective:** Immunity plays a vital role in the human papilloma virus (HPV) persistent infection, and closely associates with occurrence and development of cervical squamous cell carcinoma (CSCC). Herein, we performed an integrated bioinformatics analysis to establish an immune-gene signature and immune-associated nomogram for predicting prognosis of CSCC patients.

**Methods:** The list of immunity-associated genes was retrieved from ImmPort database. The gene and clinical information of CSCC patients were obtained from The Cancer Genome Atlas (TCGA) website. The immune gene signature for predicting overall survival (OS) of CSCC patients was constructed using the univariate Cox-regression analysis, random survival forests, and multivariate Cox-regression analysis. This signature was externally validated in GSE44001 cohort from Gene Expression Omnibus (GEO). Then, based on the established signature and the TCGA cohort with the corresponding clinical information, a nomogram was constructed and evaluated *via* Cox regression analysis, concordance index (C-index), receiver operating characteristic (ROC) curves, calibration plots and decision curve analyses (DCAs).

**Results:** A 5-immune-gene prognostic signature for CSCC was established. Low expression of *ICOS*, *ISG20* and high expression of *ANGPTL4*, *SBDS*, *LTBR* were risk factors for CSCC prognosis indicating poor OS. Based on this signature, the OS was significantly worse in high-risk group than in low-risk group (*p*-value < 0.001), the area under curves (AUCs) for 1-, 3-, 5-years OS were, respectively, 0.784, 0.727, and 0.715. A nomogram incorporating the risk score of signature and the clinical stage was constructed. The C-index of this nomogram was 0.76. AUC values were 0.811, 0.717, and 0.712 for 1-, 3-, 5-years OS. The nomogram showed good calibration and gained more net benefits than the 5-immune-gene signature and the clinical stage.

**Conclusion:** The 5-immune-gene signature may serve as a novel, independent predictor for prognosis in patients with CSCC. The nomogram incorporating the signature risk score and clinical stage improved the predictive performance than the signature and clinical stage alone for predicting 1-year OS.

## Introduction

Cervical squamous cell carcinoma (CSCC) is the major pathological type in cervical cancer (CC), accounting for approximately 75–80% of all CC (Intaraphet et al., 2013). In developing countries without a comprehensive CC screening and anti-human papilloma virus (HPV) vaccination program, the incidence and mortality of CC remains high, and the age of CC onset tends to be younger (Olson et al., 2016). Prognostic evaluation is essential for selecting patients for adjuvant therapy, setting goals for patient treatment and counselling patients (Grobmyer and Brennan, 2003). The tumor-node-metastasis (TNM) system is the most frequently utilized prognostic indicator in clinical practice. Nevertheless, the clinical TNM staging system narrowly focuses on tumor cells without considering other factors such as host immunity and inflammation responses; even patients with the same stage disease might face distinctly different outcomes (Hu et al., 2018).

It is well recognized that high-risk HPV is a key cause of CC (Evander et al., 1995; Walboomers et al., 1999). While around 90% of women contract HPV infection during their lifetimes (Rodríguez et al., 2008), only a small percentage of these infections will progress to premalignant lesion/cancer (Kulasingam et al., 2002; Dalstein et al., 2003). Recent studies pointed out that HPV infection alone is insufficient for CC; factors such as abnormal host genes (e.g., immune genes polymorphisms) and dysfunctional host immunity may also play synergistic roles in persistent HPV infection and the development of CC (Grulich et al., 2007; Wang et al., 2009; De Freitas et al., 2014; Zhang et al., 2014; Song et al., 2015). The immunity-related mechanisms behind CC remains to be further explored.

A number of immunity-related prognostic markers for CC have been identified, including *interferon gamma* (*IFNG*) (Ivansson et al., 2010), *cytotoxic T-lymphocyte associated protein 4* (*CTLA4*) (Rahimifar et al., 2010), *transporter 1, ATP binding cassette subfamily B member* (*TAP1*), and *transporter 2, ATP binding cassette subfamily B member* (*TAP2*) (Gostout et al., 2003). However, all of these are single gene markers that can be less prognostic and less stable than multi-gene signatures (Grzadkowski et al., 2018). Immune-related prognostic gene signatures based on breast cancer, lung cancer, hepatocellular carcinoma have been reported (Budhu et al., 2006; Roepman et al., 2009; Bedognetti et al., 2015). Immune-related prognostic nomogram incorporating immune gene signature and the other clinical information were also shown good performance in colorectal cancer (Song et al., 2020) and lower-grade glioma (Zhang et al., 2020). For the CC, Liu and colleagues (Liu et al., 2020) established a two-immune-gene signature (*CD6* and *CD1C*) for predicting prognosis. This signature showed good performance in TCGA cohort, even though lacking the external validation. Another study also developed a 29-immune-related gene-pairs signature for predicting the prognosis of CC (Nie et al., 2020). Nevertheless, the nomogram incorporating immune gene signature expression and other potential clinical information for evaluating the prognosis of CSCC remains to be investigated.

Given the available evidence, we proposed two hypotheses to be tested. The first hypothesis was that immune gene signature may also show prognostic significance for CSCC patients; the second was that the combination of signature and clinical information could further improve the prediction performance.

## Methods

### Data Downloads

The Gene symbol IDs of all immune-related genes were retrieved from Immunology Database and Analysis Portal (ImmPort, https://www.immport.org/home). The gene expression profiles and corresponding clinical information for CSCC patients (*n* = 254) were obtained from The Cancer Genome Atlas (TCGA, https://tcga-data.nci.nih.gov/tcga/) and further served as training dataset. In addition, the GSE44001 (*n* = 300) was retrieved from NCBI Gene Expression Omnibus (GEO, http://www.ncbi.nlm.nih.gov/geo/) and served as external validation dataset. Data were downloaded on 14 December, 2020.

### Data Preprocessing

#### Data From TCGA

The RNA-sequencing data were preprocessed following these steps:

1) Samples with overall survival (OS) of less than 30 days or with no clinical information were excluded.
2) Genes with expression levels less than one were excluded.
3) The expression profiles of immune genes were included for analysis.

#### Data From GEO

The GSE44001 data were preprocessed following these steps:

1) Probe sets were mapped to the human gene SYMBOL by the R-package ‘GEOquery’ (version 2.58). Probes without gene symbols or matching two or more gene symbols were excluded. When two or more probes matched one gene symbol, the probe with the highest value of |log fold change| was selected.
2) Genes with expression levels less than one were excluded.
3) The expression profiles of immune genes were included for analysis.

To make the expression data from two databases comparable, the “scale” function in R was used for centring and standardising.

### Construction and Evaluation of the Immune Gene Signature

After excluding 27 cases of CSCC patients with overall survival time less than 30 days, a total of 227 CSCC patients from TCGA were included in the construction of signature. Firstly, univariate Cox-regression analysis was conducted to identify survival-associated immune genes *via* R-package “survival” (version 3.2-11). Secondly, to guarantee sufficient statistical power for multivariate stepwise cox-regression, the R-package “random-ForestSRC” (version 2.9.3) (Ishwaran et al., 2008) was utilized to make a feature selection from a large number of survival-related immune genes. The random forest resembled the gating hierarchy of flow cytometry (Popescu et al., 2019). It is an ensemble classifier with a decision tree as base classifier (Cheng et al., 2018), has been well established in bioinformatics (Gosink et al., 2017). We applied the random Survival Forest (RSF) method to rank the gene importance. The genes with relative importance over 0.4 were selected in multivariate stepwise Cox-regression analysis. Based on the coefficients calculated by multivariate Cox-regression analysis, an immune-gene signature for predicting the prognosis of CSCC patients was established. Based on the expression of signature, the risk score was calculated for each case from TCGA cohort, and risk-score distribution diagrams, Kaplan–Meier (KM) survival curves, and receiver-operating characteristic (ROC) curves at the 1-, 3-, 5-years time points were conducted to evaluate the performance of the signature. We also performed KM survival curves of every single gene from the signature to explore their prognostic values. In addition, to verify the stability, this signature was further validated in the GSE44001 cohort and disease-free survival (DFS) dataset of CSCC via risk-score distribution diagrams, KM survival curves, and ROC curves.

### Construction and Evaluation of Nomogram

To better predict the prognosis of CSCC patients, a nomogram incorporating the signature risk score and other clinical characteristics was developed based on TCGA CSCC cohort. After excluding cases without detailed clinical information, a total of 220 CSCC patients were included in analysis. To be specific, variables including age, smoking status, pathological grades, clinical stages, and the signature risk score were included in the univariate Cox-regression analysis. Then, statistically significant variables were included in multivariate Cox-regression analysis, to screen independent prognostic variables for the OS of CSCC patients. The significant factors were screened based on the *p*-value < 0.05. To assess the performance (discrimination ability and calibration) of the established nomogram, concordance index (C-index) and calibration plots were utilized (Hanley and McNeil, 1982; Coutant et al., 2009; Wolbers et al., 2009). The C-index is a widely applicable measure of predictive discrimination (Harrell et al., 1996). The calibration curves were employed to compare the probability between nomogram-predicted survival with the observed survival. Moreover, the 1-, 3-, 5-years ROC curves and the 1-, 3-, 5-years decision curve analysis (DCA) (Vickers and Elkin, 2006) curves were used to compare the prediction performance of the established nomogram, immune-gene signature, and the clinical stage. DCA is a method for evaluating the clinical usefulness through quantifying net benefits at a range of threshold probabilities. Both the calibration and discrimination were evaluated through bootstrap analysis with 1,000 resamples.

### Gene Ontology and KEGG Pathway Enrichment

To explore the biological function of the immune-gene signature, GO and KEGG enrichment analysis were carried out via R-package “Clusterprofiler” (version 3.10.1) (Yu et al., 2012a), with the immune genes from the signature as gene-list and with the whole transcriptome as background. The pathway or function with adjusted *p*-value < 0.05 was selected as significantly enriched GO category/KEGG pathway.

### Gene Set Enrichment Analysis

To better understand the biological features of the immune-gene signature and every prognostic gene in this signature. Gene set enrichment analysis (GSEA) (Subramanian et al., 2005) was applied to screen significant signalling pathways based on the CSCC cohort from TCGA, with the whole transcriptome as background and with the signal-to-noise ratio as ranking criterion. Based on the median signature score or the median gene expression level (Song et al., 2019), patients were divided into two groups (i.e., high/low-risk groups classified by the established signature and high/low-expression group classified by the prognostic gene). GSEA was performed via GSEA 4.1.0 software (http://software.broadinstitute.org/gsea/). The significant pathways were screened based on false discovery rate (FDR) < 0.05.

### Immunotherapy Sensitivity Analysis

To investigate the immunotherapy sensitivity of high/low-risk groups classified by the established immune-gene signature, a published dataset containing 47 melanoma patients treated with cytotoxic T lymphocyte-associated molecule-4 (CTLA-4) and programmed cell death receptor-1 (PD-1) inhibitors (Roh et al., 2017) was used as the reference dataset as previously described (Lu et al., 2019). Then, the unsupervised Subclass Mapping (Submap) algorithm (Hoshida et al., 2007) in GenePattern (https://www.genepattern.org/) was adopted to compare the sensitivity to immune-checkpoint inhibitor therapy between high/low-risk groups classified by the signature. The Submap algorithm provided the *p*-value, demonstrating the likelihood of molecular similarity among different subclasses (Reich et al., 2006).

### Lymphocytes Infiltration Analysis

To estimate the relative abundance of 22 types of immune cells in CSCC samples, Cell type Identification By Estimating Relative Subsets Of known RNA Transcripts (CIBERSORT, https://cibersortx.stanford.edu/) was used for lymphocytes infiltration analysis. This is a method for exploring cell composition from complex tissues based on their gene expression profiles (Newman et al., 2015). The 22 immune cells include different subtypes of T cells, B cells, natural killer cells (NKs), plasma, dendritic, and mast cells, macrophages, neutrophils, and eosinophils.

### Prognostic Analysis in Gynecological Cancers

To further explore the relationship of every single gene from the immune-gene signature with the prognosis of gynecological cancers (including breast invasive carcinoma [BRCA], cervical squamous cell carcinoma and endocervical adenocarcinoma [CESC], ovarian serous cystadenocarcinoma [OV], uterine corpus endometrial carcinoma [UCEC], and uterine carcinosarcoma [UCS]), univariate survival analysis was used to analyze the correlation of patient overall survival with gene expression. Univariate Cox analysis was employed via R package “survival” (version 3.2-11).

### Statistical Analysis

R 3.4.0 software and related statistical packages were utilized for statistical analysis. Chi-square test, or Fisher-exact test (if any of the variables with value less than 5), was performed for categorical variables. The *p*-value less than 0.05 was regarded as statistically significant.

## Results

### A 5-Immune-gene Prognostic Signature was Established for CSCC Patients

**FIGURE 1:**
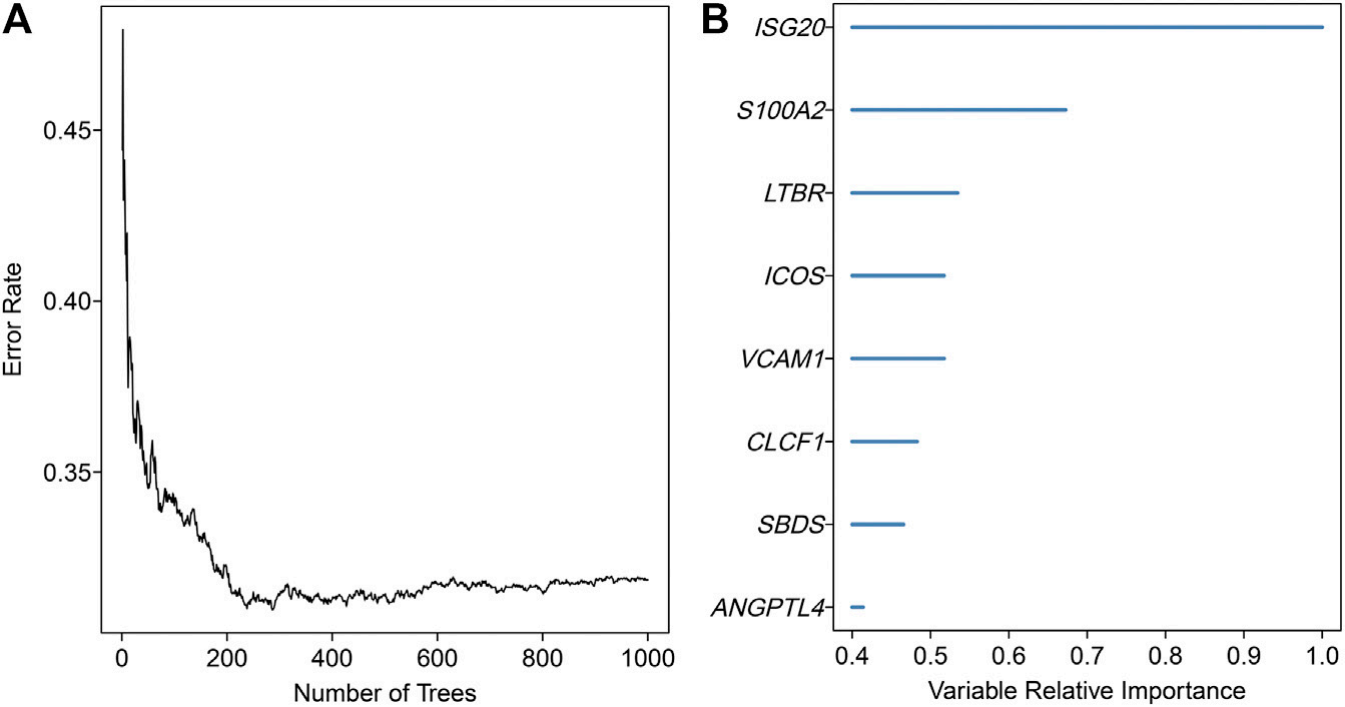
Random survival forests variable hunting analysis. **(A)** The relationship between the number of classification trees and the error rate. This random forest consisted of 1,000 decision trees and analysed all the significant survival-related genes in univariate Cox-regression analysis. **(B)** Out-of-bag importance of the survival-related genes with values over 0.4. The *x*-axis represented the relative importance values of variables estimated by random forest.

**FIGURE 2:**
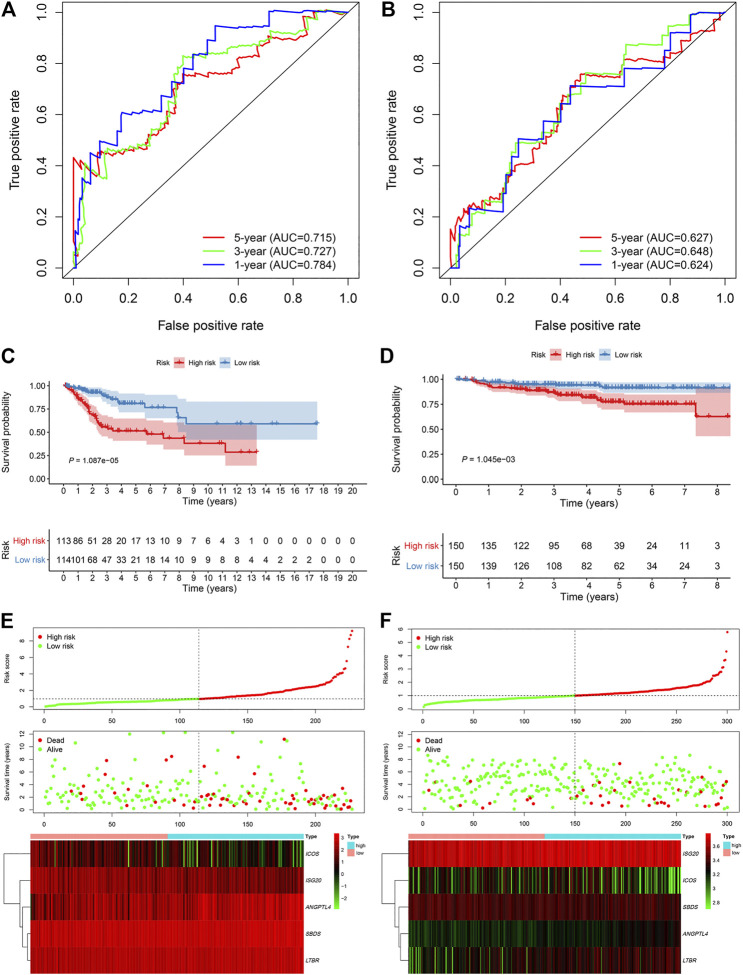
The 5-immune-gene signature showed good performance in TCGA and GEO cohort. Time-dependent ROC analysis in **(A)** TCGA cohort and **(B)** GEO cohort. Kaplan–Meier survival analysis in **(C)** TCGA and **(D)** GEO cohort. Signature risk score analysis in **(E)** TCGA and **(F)** GEO cohort. TCGA, The Cancer Genome Atlas; GEO, Gene Expression Omnibus; ROC, receiver-operating characteristic.

**FIGURE 3:**
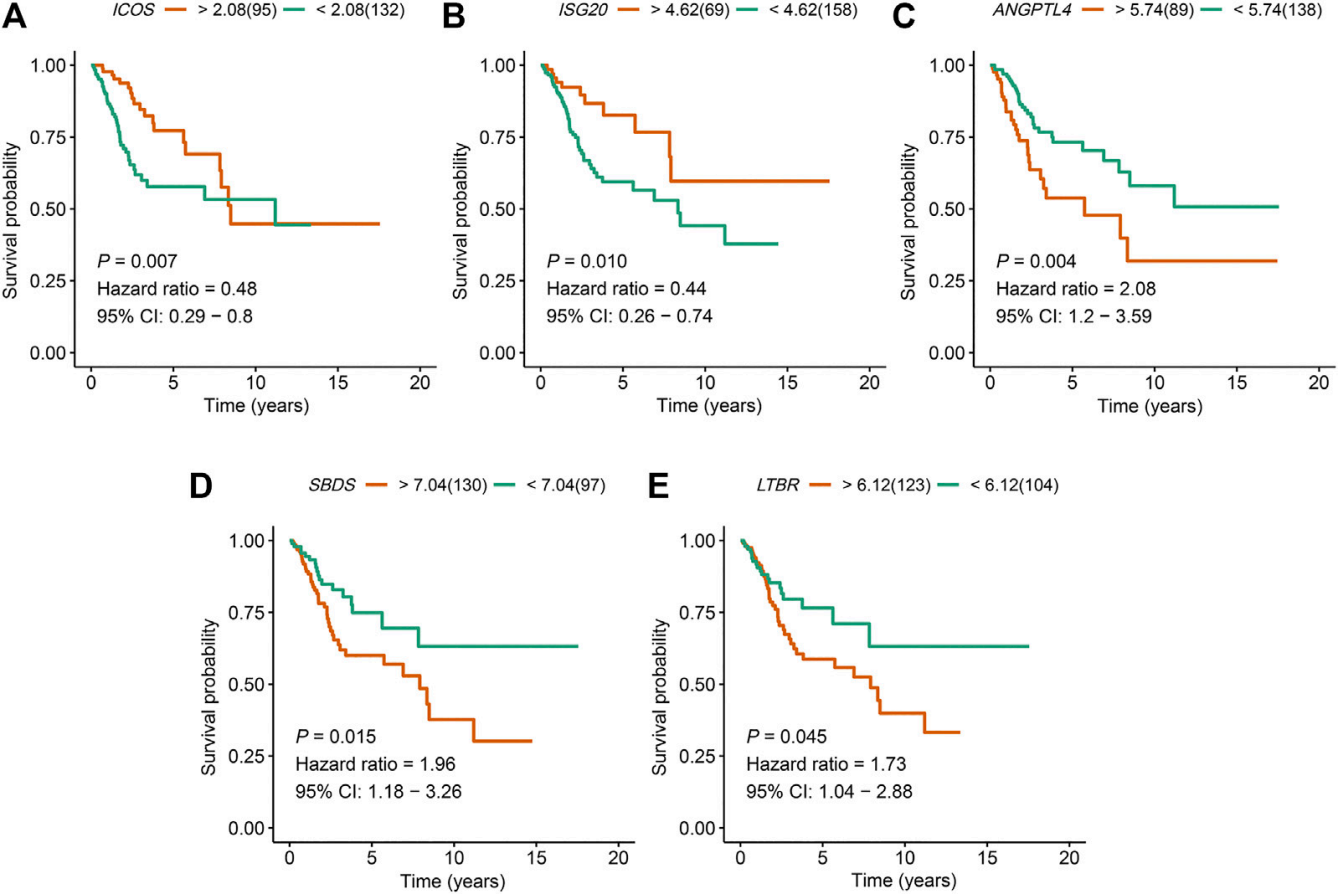
The expression of the five immune genes *ICOS*, *ISG20*, *ANGPTL4*, *SBDS*, and *LTBR* were significantly associated with the prognosis of CSCC patients. Kaplan–Meier OS curves for high and low expression of **(A)** *ICOS*, **(B)** *ISG20*, **(C)** *ANGPTL4*, **(D)** *SBDS*, and **(E)** *LTBR* in CSCC patients. *ICOS*, *Inducible T Cell Costimulator*; *ISG20*, *Interferon Stimulated Exonuclease Gene 20*; *ANGPTL4*, *Angiopoietin Like 4*; *SBDS*, *SBDS Ribosome Maturation Factor*; *LTBR, Lymphotoxin Beta Receptor*; CSCC, cervical squamous cell carcinoma; OS, overall survival.

An overview of the analysis procedure of the current study was shown in Supplementary Figure S1. The random forest analysis was performed and identified eight survival-related immune genes with out-of-bag importance over 0.4 (Figure 1). After multivariable stepwise regression analysis based on the eight survival-related immune genes, a 5-immune-gene prognostic signature was finally constructed. These genes included *Inducible T Cell Costimulator* (*ICOS*, average TPM: 3.711), *Interferon Stimulated Exonuclease Gene 20* (*ISG20*, average TPM: 19.819), *Angiopoietin Like 4* (*ANGPTL4*, average TPM: 72.436), *SBDS Ribosome Maturation Factor* (*SBDS*, average TPM: 150.027), *Lymphotoxin Beta Receptor* (*LTBR*, average TPM: 79.407). For each individual CSCC patient, a signature risk score was calculated *via* the following formula: Score = *ICOS* × (−0.34244) + *ISG20* × (−0.24956) + *ANGPTL4* × 0.270329 + *SBDS* × 0.423242 + *LTBR* × 0.401314. Patients were divided into high/low-risk groups *via* the median risk score (0.971). The clinical characteristics of the high/low-risk groups in TCGA dataset were shown in Table 1. Based on the 5-immune-gene signature, the area under curves (AUCs) for 1-, 3-, and 5-years OS were respectively 0.784, 0.727, and 0.715 (Figure 2A). Compared with the low-risk group, the high-risk group showed significantly worse OS (*p*-value < 0.001; Figures 2C,E). This 5-immune-gene signature was externally validated in the GEO cohort with DFS information. The clinical information of the GEO cohort was shown in Table 1. The AUCs were 0.624 at 1 year, 0.648 at 3 years, and 0.627 at 5 years. (Figure 2B). Compared with low-risk group, the high-risk group of the GEO cohort had significantly worse probability of DFS (*p*-value = 0.001; Figures 2D,F). The 5-immune-gene signature was also evaluated in the TCGA cohort with DFS information. The AUC of DFS was 0.695 (Supplementary Figure S2A), the high-risk group had significantly worse probability of DFS than low-risk group (*p*-value = 0.043; Supplementary Figures S2B,C). Taken together, the 5-immune-gene signature exhibited good performance of prognostic prediction. In this signature, low expression levels of *ICOS* (HR = 0.48, 95% CI = 0.29–0.80, *p*-value = 0.007), *ISG20* (HR = 0.44, 95% CI = 0.26–0.74, *p*-value = 0.010) and high expression levels of *ANGPTL4* (HR = 2.08, 95% CI = 1.20–3.59, *p*-value = 0.004), *SBDS* (HR = 1.96, 95% CI = 1.18–3.26, *p*-value = 0.015), and *LTBR* (HR = 1.73, 95% CI = 1.04–2.88, *p*-value = 0.045) were risk factors for CSCC prognosis, suggesting poor OS (Figures 3A–E).

**TABLE 1:** Clinical characteristics of TCGA and GEO cohorts.

TCGA cohort				
	High-risk (*n* = 113)	Low-risk (*n* = 114)	Total (*n* = 227)	*p*-value
Age (years)				
＜65	96	101	197	0.539
≥65	17	13	30	
Clinical stage				
Stage I	63	52	115	0.090
Stage II	19	35	54	
Stage III	19	17	36	
Stage IV	9	6	15	
N/A	3	4	7	
T Stage				
T1	72	55	127	0.056
T2	21	38	59	
T3	6	8	14	
T4	5	4	9	
TX	9	9	18	
M Stage				
M0	51	47	98	0.270
M1	2	6	8	
MX	60	61	121	
N stage				
N0	59	62	121	0.362
N1	30	22	52	
NX	24	30	54	
Pathological grade				
G1	6	4	10	0.539
G2	47	56	103	
G3	48	45	93	
G4	0	1	1	
GX	12	8	20	
**GEO cohort**				
	**High-risk (n = 150)**	**Low-risk (n = 150)**	**Total (n = 300)**	***p*-value**
Clinical stage				
Stage I	127	131	258	0.618
Stage II	23	19	42	

T, tumor size; M, distant metastasis; N, lymph node metastasis. The p-values were determined by comparison between the high- and low-risk groups.

### Prognostic Nomogram Incorporating Signature Risk Score and Clinical Stage was Constructed for CSCC Patients

**FIGURE 4:**
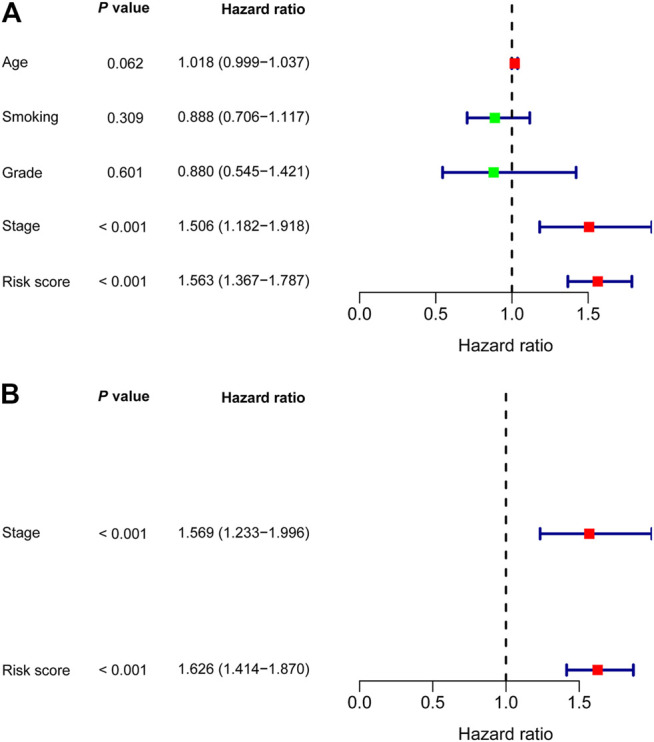
Nomogram was developed based on **(A)** Univariate and **(B)** multivariate Cox regression analysis.

**FIGURE 5:**
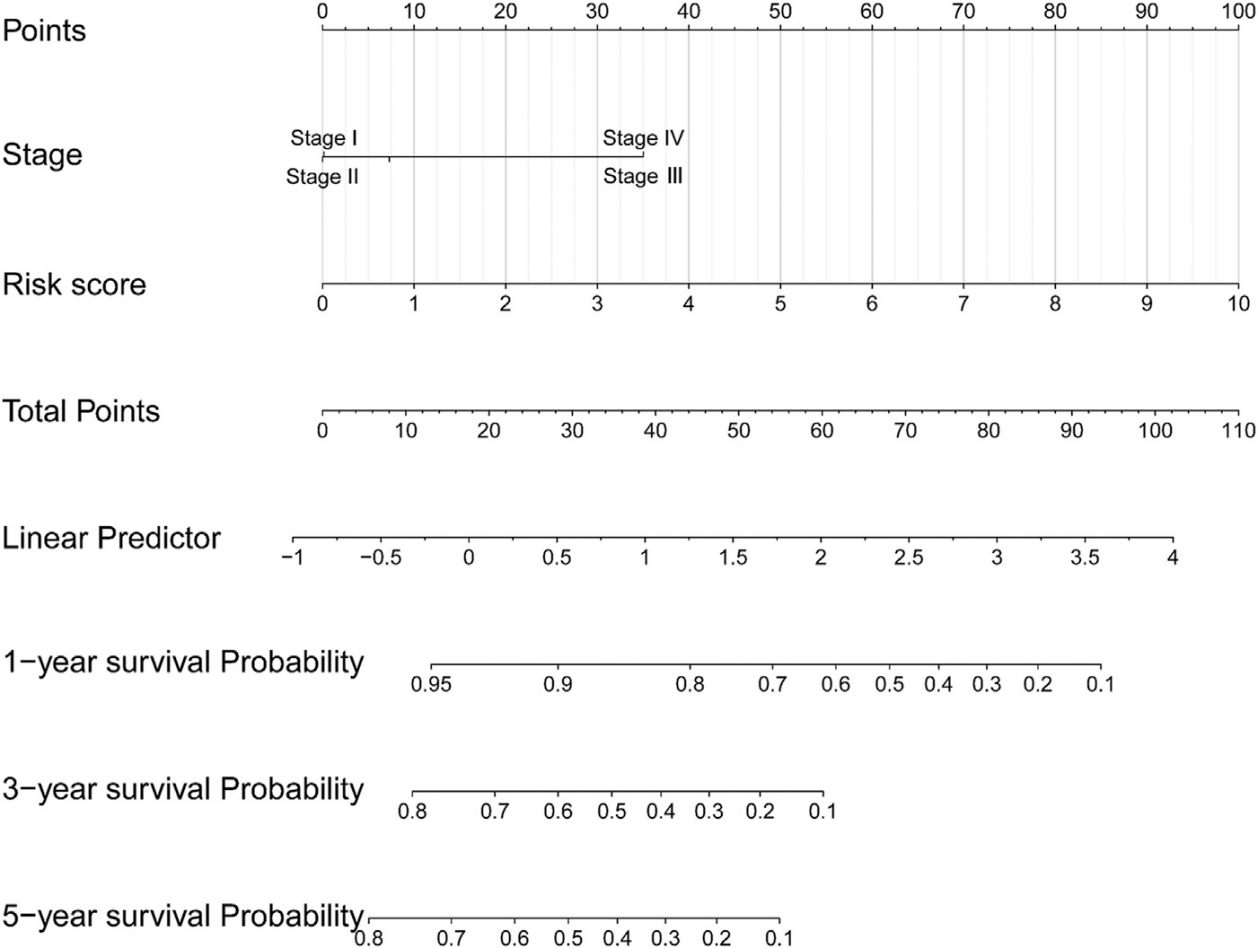
A nomogram incorporating 5-immune-gene signature and clinical stage was constructed for predicting OS of CSCC patients.

**FIGURE 6:**
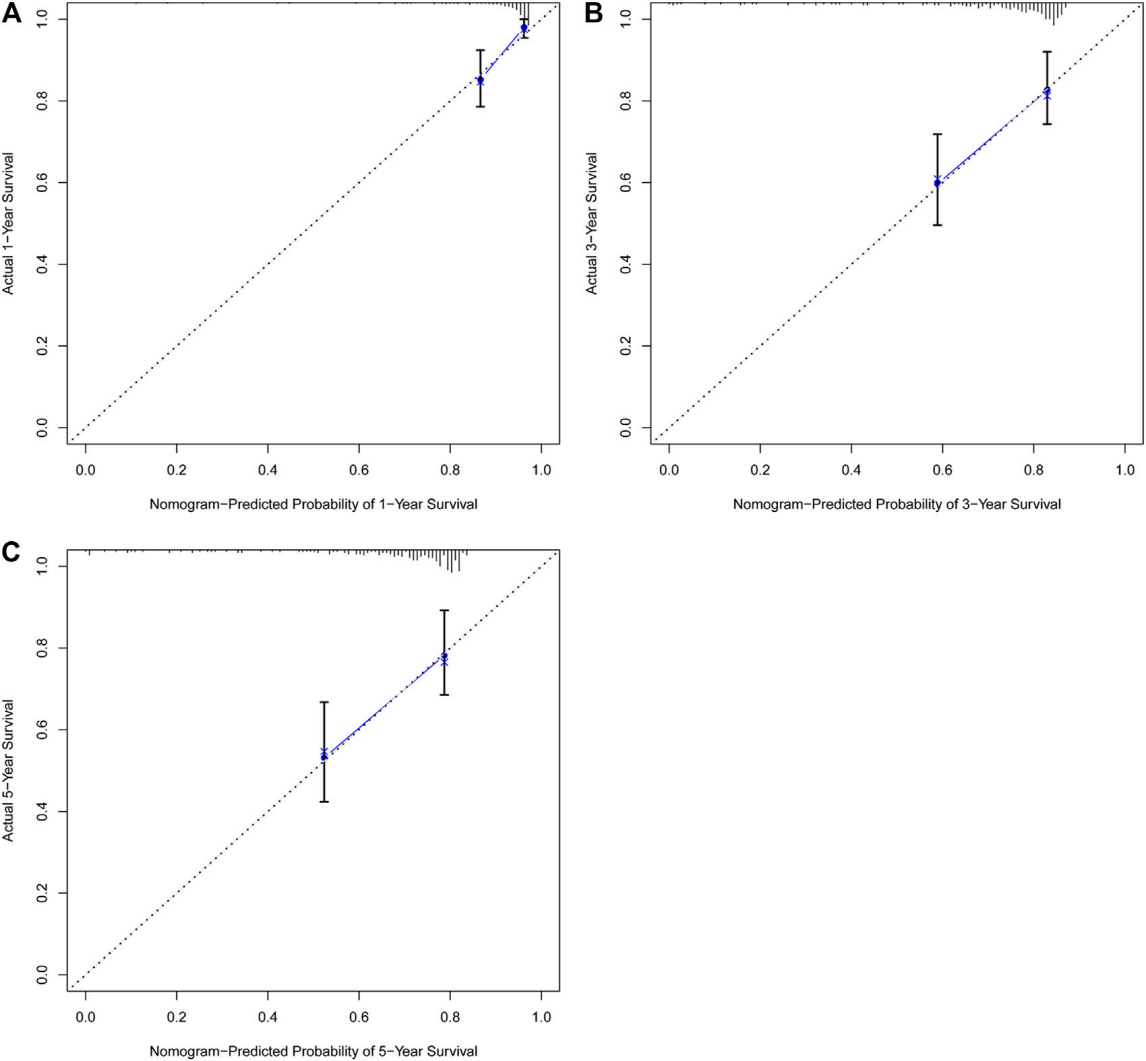
The nomogram showed favorable agreement between nomogram-predicted OS probability and actual OS probability. Calibration plots for predicting **(A)** 1-year, **(B)** 3-years, and **(C)** 5-years OS for CSCC patients based on the nomogram. The 45-degree dotted line represents the “ideal” prediction. The blue line represents the performance of the nomogram. The blue line closer to the ideal prediction has a higher predictive accuracy of the nomogram.

**FIGURE 7:**
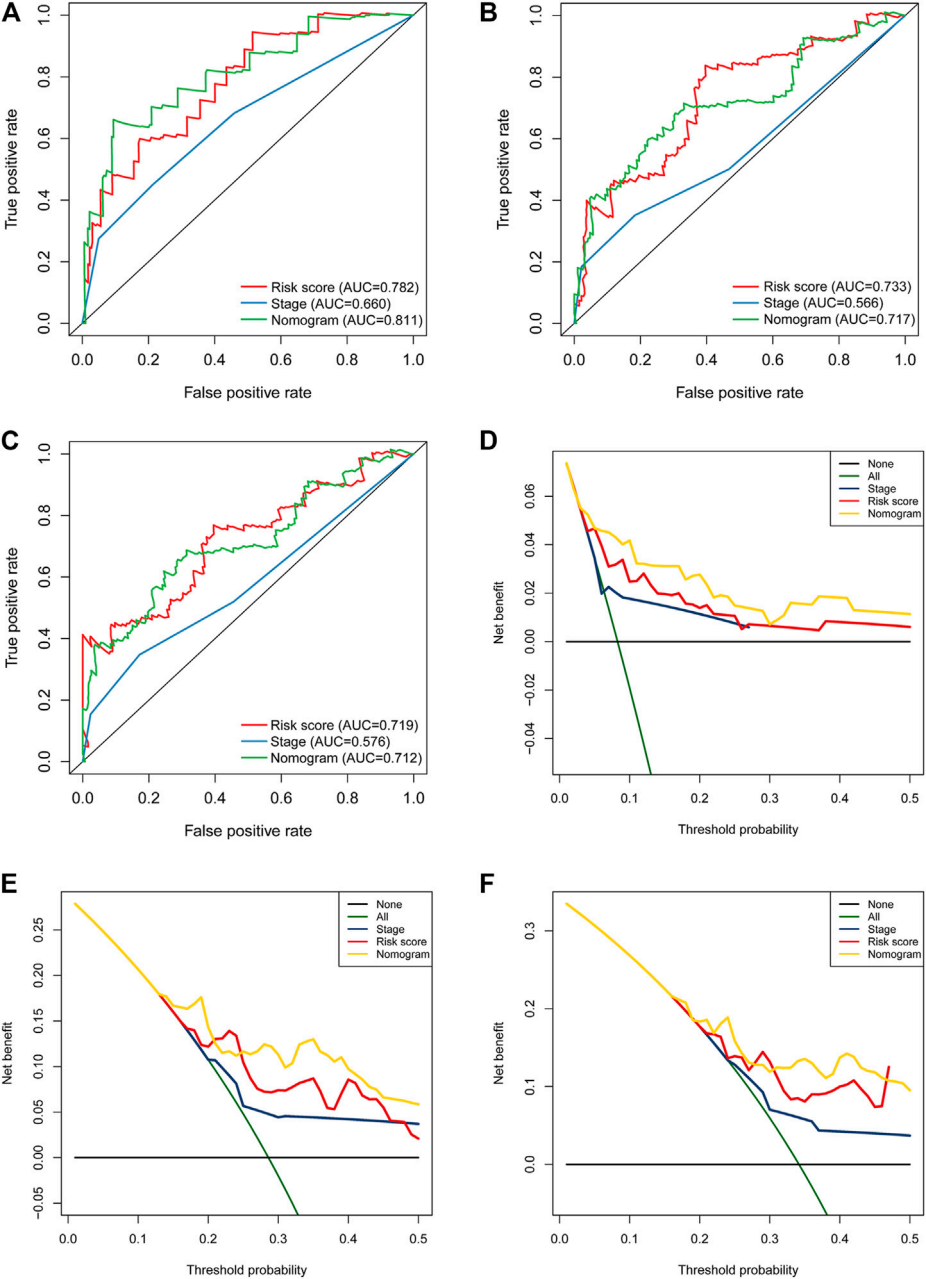
The nomogram showed better predictive power than the 5-immune-gene signature and clinical stage for 1-year OS. Time-dependent ROC curves of the nomograms, 5-immune-gene signature, and clinical stage in prediction of **(A)** 1-year, **(B)** 3-years, and **(C)** 5-years OS for CSCC patients. DCA curves for the nomogram, 5-immune-gene signature, and clinical stage in prediction of **(D)** 1-year, **(E)** 3-years, and **(F)** 5-years OS for CSCC patients. DCA, decision curve analysis.

In univariate Cox regression analysis (Figure 4A), the 5-immune-gene signature risk scores (HR = 1.56, 95% CI = 1.37–1.79, *p*-value < 0.001) and the clinical stages (HR = 1.51, 95% CI = 1.18–1.92, *p*-value < 0.001) were significantly correlated with the OS in CSCC patients. In multivariate Cox regression analysis (Figure 4B), the signature risk scores (HR = 1.63, 95% CI = 1.41–1.87, *p*-value < 0.001) and the clinical stages (HR = 1.57, 95% CI = 1.23–2.00, *p*-value < 0.001) turned out to be the independent predictors for the OS of CSCC patients. We further constructed and evaluated a nomogram (Figure 5) incorporating signature risk score and clinical stage. The nomogram had favorable discrimination (C-index = 0.76; 95% CI = 0.70–0.82). The calibration curves of the nomogram showed favorable agreement between actual OS probability and nomogram-predicted OS probability (Figures 6A–C). When compared with 5-immune-gene signature and clinical stage, the nomogram (AUC = 0.811) showed better predictive power than signature risk score (AUC = 0.782) and clinical stage for 1-year OS (AUC = 0.660; Figure 7A). For the OS at 3- and 5-years, the predictive power of the prognostic nomogram was similar to that of the signature risk score and better than that of clinical stage (AUC at 3 years = 0.566, AUC at 5 years = 0.576, Figures 7B,C). The DCA curves at 1-, 3-, and 5- years revealed that the nomogram had more net benefits than the signature risk score and the clinical stage (range: 0–0.5; Figures 7D–F).

### Enrichment Analysis Based on the 5-Immune-gene Signature

**FIGURE 8:**
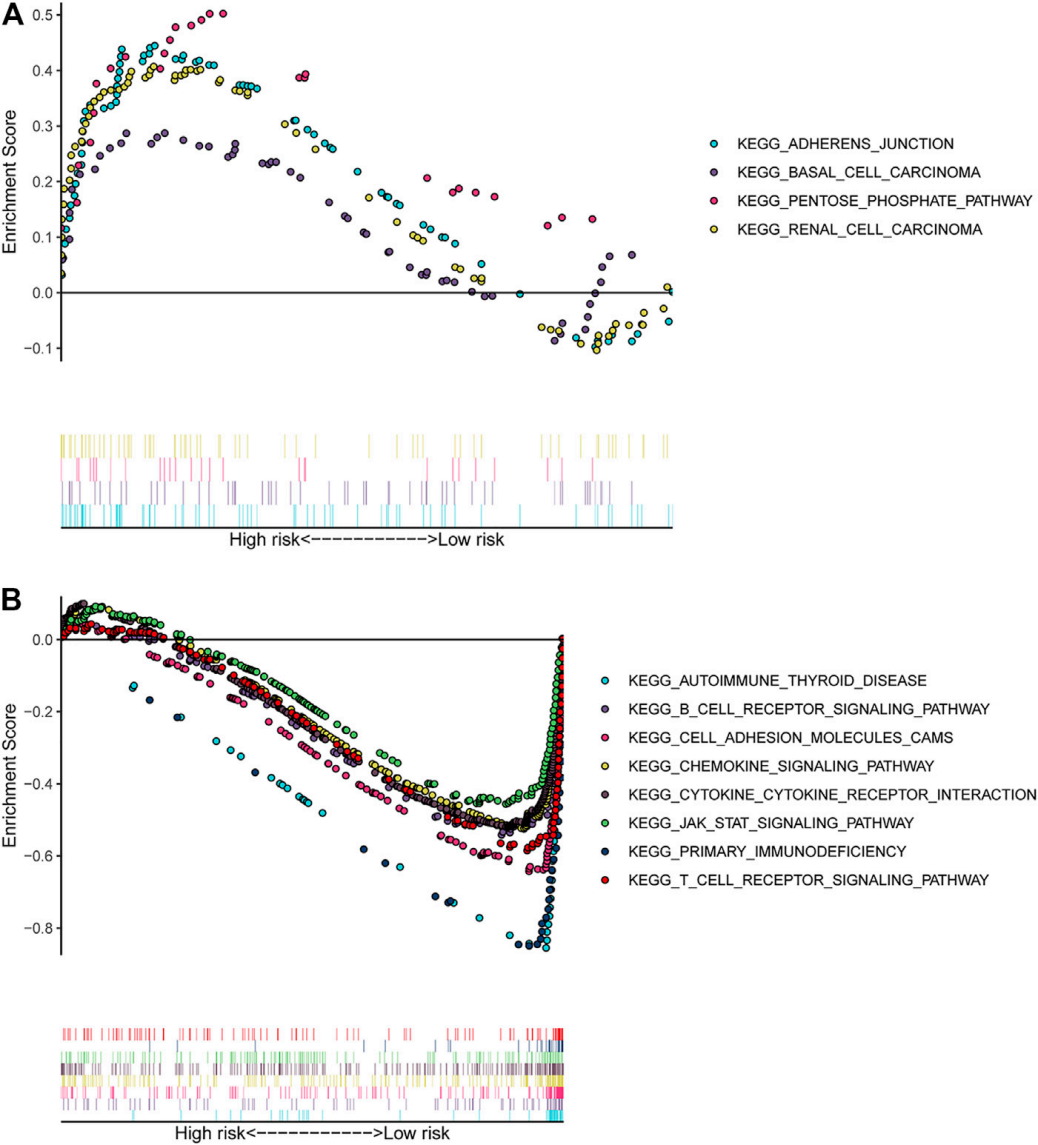
GSEA analysis based on the 5-immune-gene signature. **(A)** Significantly enriched pathways in the high-risk group of CSCC; **(B)** significantly enriched pathways in the low-risk group of CSCC. The *y*-axis represented the enrichment score calculated by GSEA. Vertical bars under the *x*-axis represented the positions of genes in the given set. Positive enrichment scores denoted the pathways were significantly enriched in high-risk group, while negative enrichment scores denoted the pathways were significantly enriched in low-risk group. GSEA, gene set enrichment analysis.

The GSEA analysis result based on the signature revealed that “adherens junction,” “basal cell carcinoma,” “pentose phosphate pathway,” and “renal cell carcinoma” signalling pathways were significantly enriched (*p*-value < 0.05) in the high-risk group of CSCC patients (Figure 8A). The “B cell receptor signalling pathway,” “primary immunodeficiency,” and “T cell receptor signalling pathway” were significantly enriched (*p*-value < 0.05) in the low-risk group (Figure 8B).

GO analysis revealed that the signature was mainly enriched in “T cell activation” for biological process (BP) (Supplementary Figure S3). The KEGG pathway analysis suggested that the signature was predominantly enriched in “T cell receptor signalling pathway,” “Th1, Th2, and Th17 cell differentiation,” and “PD-L1 expression and PD-1 checkpoint pathway in cancer” (Supplementary Figure S4).

### Immunotherapy Sensitivity Analysis Based on the 5-Immune-gene Signature

**FIGURE 9:**
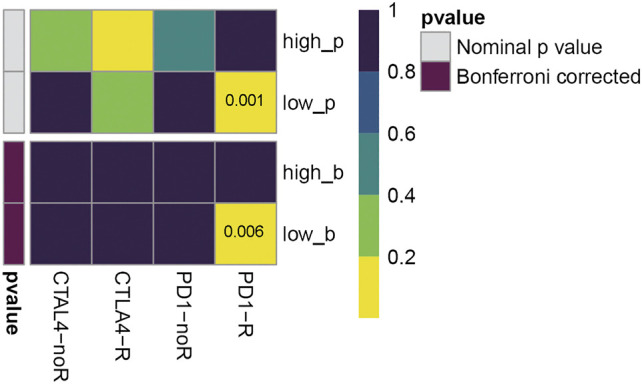
Effect prediction of immunotherapy response in TCGA cohort. The horizontal axis shows the groups placed on CTLA-4 and PD-1 immunotherapies. PD1-R, patients respond to PD-1 therapy, PD1-noR, patients do not respond to PD-1 therapy. The vertical axis shows the high/low-risk groups classified by five-gene prognostic signature, p refers to nominal *p*-value, b refers to Bonferroni-corrected *p*-value. The color represents the significance levels (*p*-values).

To further investigate the clinical implication of 5-immune-gene signature, we used SubMap algorithm to compare the immunotherapy sensitivity between high/low-risk groups classified by this signature. As shown in Figure 9, the low-risk group was more likely to respond to PD-1 checkpoint immunotherapies (Bonferroni-corrected *p*-value = 0.006).

### Lymphocytes Infiltration and GSEA and Gynecological-Cancer Prognostic Analysis Based on the Five Immune Genes

The correlation between the five immune genes and the infiltration of 22 lymphocytes was shown in Supplementary Figure S5. This analysis demonstrated that these five genes were all significantly associated with the infiltration of one or more lymphocytes of 22 lymphocytes. It should be noted that both of the *ISG20* and *ICOS* showed negative correlations with “naïve B cells,” “Eosinophils,” “M0 Macrophages,” and “CD4+ resting memory T cells,” and showed positive correlations with “activated memory CD4+ T cells,” and “CD8+ T cells” (*p*-value < 0.05). The *SBDS* and *ANGPTL4* were positively correlated with “activated mast cells”, and *ICOS* was negatively correlated with “activated mast cells” (*p*-values < 0.05).

Supplementary Figures S6–8 showed the GSEA results for each prognostic gene with statistical significance. GSEA of *ICOS* (Supplementary Figure S6) revealed that “T cell receptor signalling pathway” was significantly enriched in *ICOS*-high-expressing group (*p*-value < 0.05). GSEA of *LTBR* (Supplementary Figure S8) found that “galactose metabolism” pathway was significantly enriched in *LTBR*-high-expressing group (*p*-value < 0.05).

As shown in Supplementary Figure S9, in terms of overall survival, not only CESC patients, but also OV patients, were significantly associated with *ICOS*, *ISG20*, *ANGPTL4*, and *SBDS* (all *p*-values < 0.05).

## Discussion

In this study, to predict prognosis of CSCC patients, we established a 5-immune-gene signature (including *ICOS, ISG20, ANGPTL4, SBDS, and LTBR*) in TCGA cohort and externally validated it in GEO cohorts. Based on the 5-immune-gene signature, we constructed a nomogram incorporating signature risk score and clinical stage. This nomogram showed good accuracy for predicting overall survival of CSCC patients (1-, 3-, 5-years AUC were 0.811, 0.717, and 0.712, respectively).

Among the five immune genes from the established signature, several genes have been explored in previous studies. *ISG20* is an RNA exonuclease that exhibits inhibitory activity against various viruses (e.g., human immunodeficiency virus (Espert et al., 2005), influenza virus, vesicular stomatitis virus, and encephalomyocarditis virus (Zhou et al., 2011)). In our analysis, downregulation of *ISG20* was associated with poor outcome of CSCC. This result appears to be in discordance with that of Rajkumar et al. (2011), who found that *ISG20* was up-regulated in CC and may get involved in the progression of cervical disease (Rajkumar et al., 2011). But to be noted, Rajkumar et al. (2011) included all pathological types of CC, the sample size was limited (*n* = 28), follow-up information of CC patients was lacking, and up-regulation of *ISG20* was regarded as being involved in cervical tumorigenesis, not for predicting outcome.

We also found that *ANGPTL4* (angiopoietin-like protein 4) was up-regulated in CSCC tissues with poor prognosis. This finding is in accordance with that of Nie et al., who reported that *ANGPTL4* was up-regulated in CC samples, and this up-regulation was associated with lymph node metastasis, deep stromal invasion, lympho-vascular space invasion, and advanced tumor stage, as well as poor OS and DFS (Nie et al., 2019). *ANGPTL4* is a member of the angiopoietin-related family, and involves the regulation of glucolipid and angiogenesis metabolism (Hato et al., 2008; Li and Teng, 2014). The up-regulated *ANGPTL4* contributed to tumor angiogenesis, growth, metastasis, invasion, and reduced OS of patients (Shibata et al., 2010; Li et al., 2011; Tanaka et al., 2015; Huang et al., 2016; Zhu et al., 2016). Meanwhile, *ANGPTL4* polymorphism was found to be associated with cervical neoplasia development (Rahmani et al., 2020). For patients with CC, *ANGPTL4* might serve as a marker for poor survival (Nie et al., 2019). Nevertheless, there remains a need to elucidate the exact tumorigenesis mechanism of *ANGPTL4* in CC.

*SBDS* encodes a highly conserved protein in archaebacteria and eukaryotes (Boocock et al., 2003). It is multifunctional, involved in ribosome synthesis, chemotaxis, telomerase recruitment, assembly of mitotic spindle, and regulation of reactive oxygen species generation (Wessels et al., 2006; Austin et al., 2008; Ambekar et al., 2010; Spalinger et al., 2018). The role of *SBDS* in cancer is not well clarified. In the current analysis, the *SBDS* was highly expressed in CSCC tissues and predicted worse OS. This finding accords with those of Hao et al. (2020). But, to be noted, Hao et al. (2020) also found that *SBDS* played dual roles in cancer development (i.e., both tumor-promoting and tumor-suppressive roles). *SBDS* is essential for ribosome assembly in cytoplasm and rRNA synthesis in nucleolus, which may confer its oncogenic action in cancer. By contrast, ectopic SBDS in the nucleoplasm may exert a tumor-suppressive effect by activating p53 (Ganapathi et al., 2007; Finch et al., 2011; Hao et al., 2020).

The associations between *ICOS, LTBR* with CSCC prognosis have not been completely clarified; however, one study reported that *ICOS* gene polymorphisms showed no association with susceptibility to CSCC (Pawlak et al., 2010). Our study revealed that low expression of *ICOS* was risk factor for CSCC prognosis. Moreover, we found the *ICOS* expression was associated with the prognosis of CESC, OV, and UCEC. Based on the GSEA of *ICOS* and the enrichment analysis of the 5-immune-gene signature (i.e., GO, KEGG, and GSEA), it seems that *ICSO* combining with other four immune genes may enrich in the pathway of T cell receptor and play a role in regulating the activation of T cell. In the cancer immunotherapy, T cell activation is critical for therapeutic efficacy (Nam et al., 2018). The well-known PD-1 inhibitor is designed to interrupt the interactions of PD-1 receptor with ligand and thus restore the anti-tumor responses from T cell (Agulló-Ortuño et al., 2020).

In addition, through the submap algorithm, we were delighted to see the low-risk group classified by the 5-immune-gene signature was more likely to benefit from PD-1 checkpoint immunotherapies. Over the past decades, immunotherapies have dramatically changed the treatment algorithm for melanoma. Immune-checkpoint inhibitors (Schumacher and Schreiber, 2015), particularly PD-1/programmed cell death ligand-1(PD-L1) inhibitors (Constantinidou et al., 2019), have exhibited favorable efficacy against various solid tumors (Gettinger et al., 2018). It has also been revealed that the inhibitors of PD-1/PD-L1 might be a promising method for treatment for CC (Liu et al., 2019), while the reported response rates of PD-1/PD-L1 inhibitors against recurrent or metastatic CC were under 30% (Frenel et al., 2017; Hollebecque et al., 2017; Schellens et al., 2017). Cancer patients’ response to immunotherapies remains a challenging field of research. Our study found that the 5-immune-gene signature was correlated with response to PD-1 inhibitors, indicating this 5-immune-gene signature may help doctors to screen for patients who have more potential to respond to anti-PD-1 immunotherapy, achieving more accurate and personalised treatment plans.

Based on the CIBERSORT algorithm, the current analysis found that expression of all five genes was significantly correlated with the lymphocyte infiltration. These results reveal that the five immune genes play significant roles in the immune microenvironment, and the dysregulated immune systems may be involved in the progression of CSCC. “Activated mast cells” was positively associated with *SBDS* and *ANGPTL4* but negatively associated with *ICOS,* suggesting that the infiltration levels of activated mast cells (MCs) were increased in the high-risk group of CSCC patients. This is in accordance with the study of Wang et al., who found that the higher fraction of activated MCs were independent risk factors for CC, indicating worse OS. MCs have been found to be involved in inflammatory, immune reactions, angiogenesis, tissue remodelling, immune-modulation, and carcinogenesis of various cancers, including CC (Maltby et al., 2009; Utrera-Barillas et al., 2010). In cervical tissues with intra-epithelial lesions or invasive lesions, microvessel density significantly correlated with the density of MCs (Wilk et al., 2010). MCs play several roles in both physiological and pathological processes. Normally, MCs are located at the boundary of the external environment, where they serve as sentinels for pathogen invasion and tissue damage (Galli and Tsai, 2012); however, when MCs become over-activated, such as during systemic or chronic infections, they often cause pathogenic sequelae (Choi et al., 2020). One study found both a pro-tumorigenic and anti-tumorigenic role of MCs (Varricchi et al., 2017a). Overall, the impacts of MCs are complicated and controversial, deserving further investigation (Theoharides and Conti, 2004; Varricchi et al., 2017b).

The potential study limitations: in this study, we adopted the random forest and COX regression analysis to select hub genes for the prognostic signature, and established a 5-immune-gene signature with good performance; however, due to the limited sample size of the study cohort and the complex nature of biology, we could not guarantee good stability of this signature in the real-world cohort. Despite the fact that an external GEO data-set of gene expression profiles of CSCC was obtained for validation, it remains essential that the findings of the current study should be validated in large-scale clinical cohort studies. For the future studies aiming at the establishment of prognostic signature, not only the mechanisms of the feature selection methods, but also the sample size and the data distribution should be considered (Yu et al., 2012b).

## Conclusion

In summary, we established an immune gene-based signature for predicting prognosis of CSCC patients; besides, this signature could also help in predicting response to anti-PD-1 immunotherapy. Further, we developed a nomogram combining the signature and clinical stage, which showed better prediction performance than the individual signature and clinical stage. Our findings give the important indication that the immune gene-based classifier should prove valuable in prognosis prediction and clinical decision-making for CSCC patients.

## Data Availability

The datasets presented in this study can be found in online repositories. The names of the repository/repositories and accession number(s) can be found in the article/Supplementary Material.

## Supplementary Material

Supplementary Figure 1 Flowchart of this study. TCGA, The Cancer Genome Atlas; GEO, Gene Expression Omnibus; CESC, cervical squamous cell carcinoma and endocervical adenocarcinoma; CSCC, cervical squamous cell carcinoma; DFS, disease-free survival; GO, Gene Ontology; GSEA, gene set enrichment analysis; ROC, receiver-operating characteristic; DCA, decision curve analysis; *ICOS*, *Inducible T Cell Costimulator*; *ISG20*, *Interferon Stimulated Exonuclease Gene 20*; *ANGPTL4*, *Angiopoietin Like 4*; *SBDS*, *SBDS Ribosome Maturation Factor*; *LTBR, Lymphotoxin Beta Receptor.*

Supplementary Figure 2 Prognostic value of the 5-immune-gene signature for predicting DFS of TCGA CSCC patients. **(A)** ROC analysis, **(B)** Kaplan–Meier survival analysis and **(C)** signature risk score analysis.

Supplementary Figure 3 GO enrichment analysis of the 5-immune-gene signature. The y-axis represented the top 10 most significant terms in BP, CC and MF; the x-axis represented counts of genes enriched in GO terms. The color gradient from red to blue meant the adjusted *p*-value from low to high. BP, biological process; CC, cellular component; MF, molecular function.

Supplementary Figure 4 KEGG pathway enrichment analysis of the 5-immune-gene signature. The y-axis represented the top 30 most significant KEGG terms; the x-axis represented counts of genes enriched in KEGG terms. The color gradient from red to blue meant the adjusted *p*-value from low to high.

Supplementary Figure 5 Lymphocyte infiltration analysis. The association between the five immune genes and infiltration of 22 lymphocytes in CSCC samples. Red, positive correlation; blue, negative correlation.

Supplementary Figure 6 Single-gene GSEA analysis of *ICOS* discovered seven pathways significantly upregulated in the *ICOS*-high-expressing group. The y-axis represented the enrichment score calculated by GSEA. Vertical bars under the x-axis represented the positions of genes in the given set. Positive enrichment scores denoted the pathways were significantly enriched in *ICOS*-high-expressing group, while negative enrichment scores denoted the pathways were significantly enriched in *ICOS*-low-expressing group.

Supplementary Figure 7 Single-gene GSEA analysis of *ISG20* discovered seven pathways significantly upregulated in the *ISG20*-high-expressing group. The y-axis represented the enrichment score calculated by GSEA. Vertical bars under the x-axis represented the positions of genes in the given set. Positive enrichment scores denoted the pathways were significantly enriched in *ISG20*-high-expressing group, while negative enrichment scores denoted the pathways were significantly enriched in *ISG20*-low-expressing group.

Supplementary Figure 8 Single-gene GSEA analysis of *LTBR* discovered one pathway significantly upregulated in the *LTBR*-high-expressing group. The y-axis represented the enrichment score calculated by GSEA. Vertical bars under the x-axis represented the positions of genes in the given set. Positive enrichment scores denoted the pathways were significantly enriched in *LTBR*-high-expressing group, while negative enrichment scores denoted the pathways were significantly enriched in *LTBR*-low-expressing group.

Supplementary Figure 9 Forest plot of the relationship between *ICOS*, *ISG20*, *ANGPTL4*, *SBDS*, and *LTBR* expression with the overall survival of gynecological cancers. BRCA, breast invasive carcinoma; CESC, cervical squamous cell carcinoma and endocervical adenocarcinoma; OV, ovarian serous cystadenocarcinoma; UCEC, uterine corpus endometrial carcinoma; UCS, uterine carcinosarcoma; HR, hazard ratio.
