# Supplementary figures and images for: An Immune-Gene-Based Classifier Predicts Prognosis in Patients With Cervical Squamous Cell Carcinoma
Source: Front Mol Biosci. 2021 Jul 5;8:679474. doi: 10.3389/fmolb.2021.679474 (PMC8289438; doi:10.3389/fmolb.2021.679474)

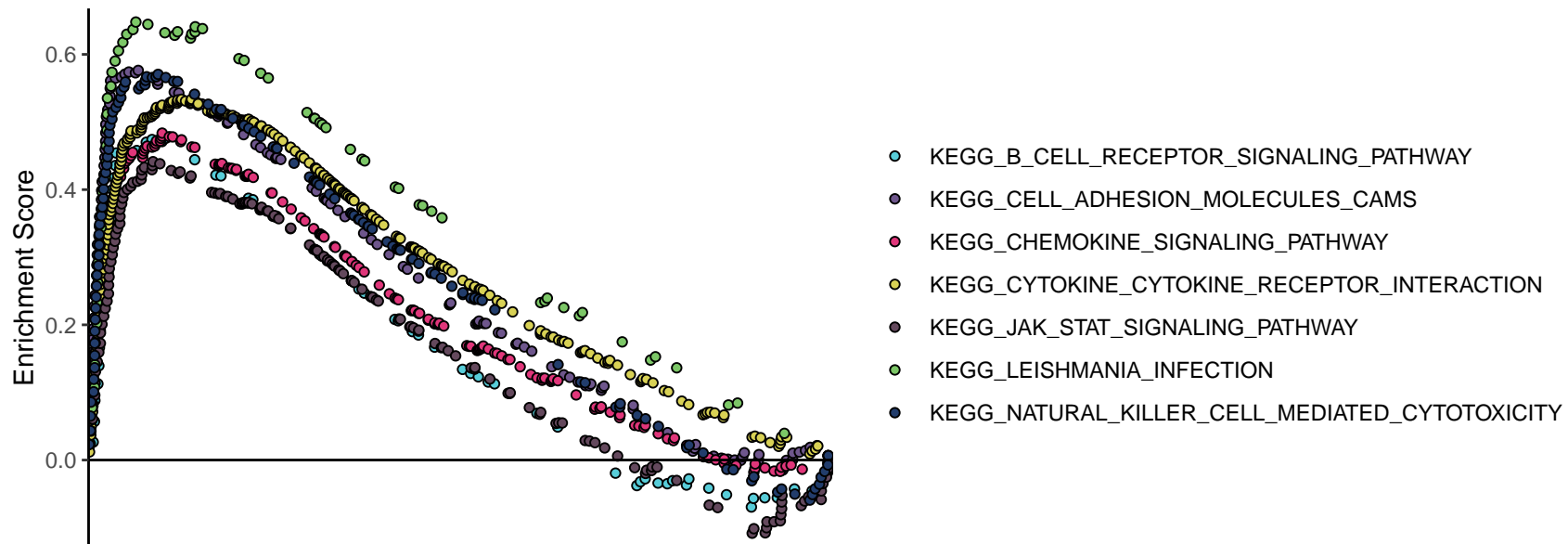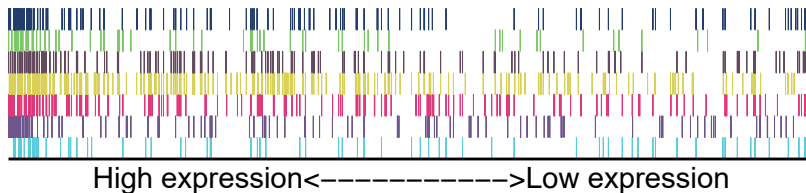

Supplement: Supplementary file 1 [file DataSheet7.PDF]

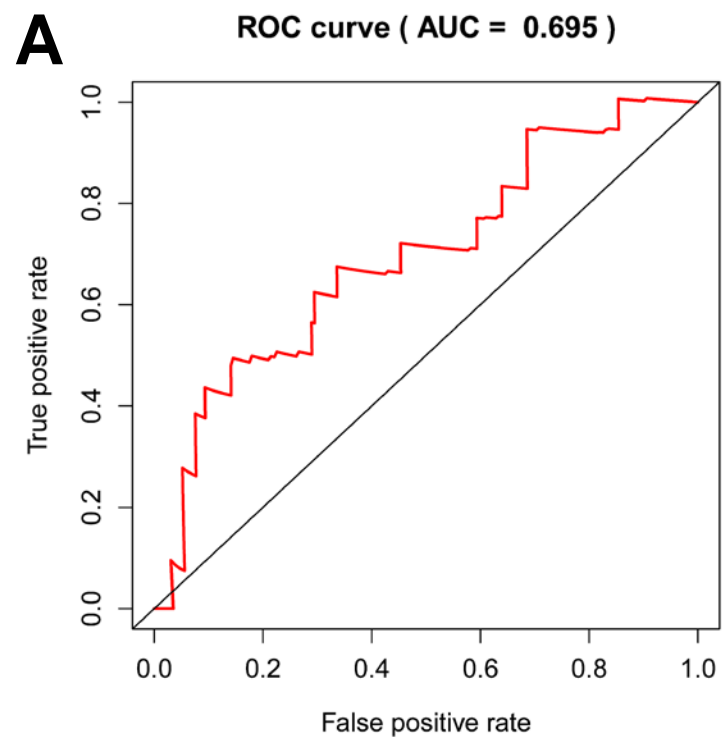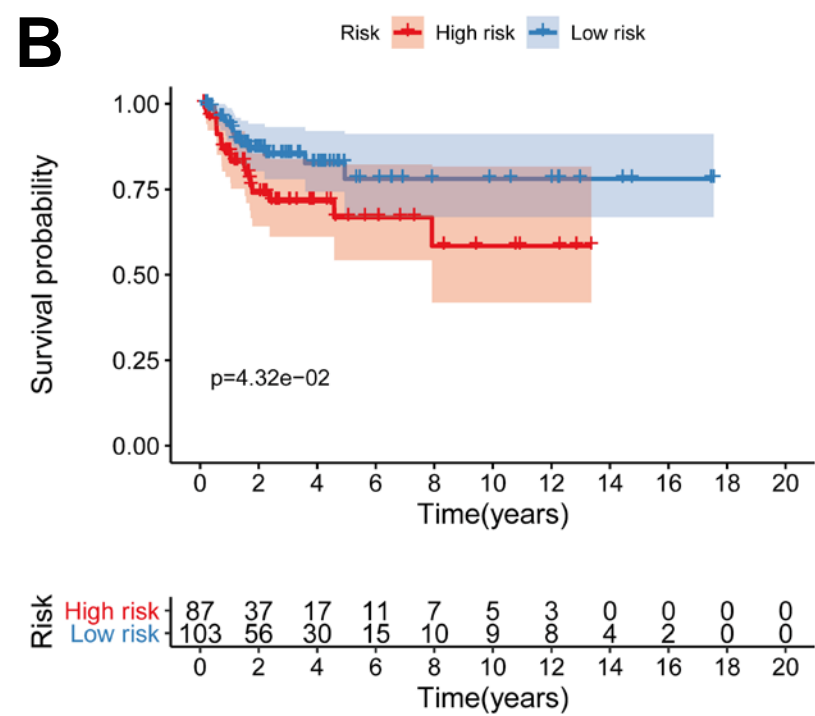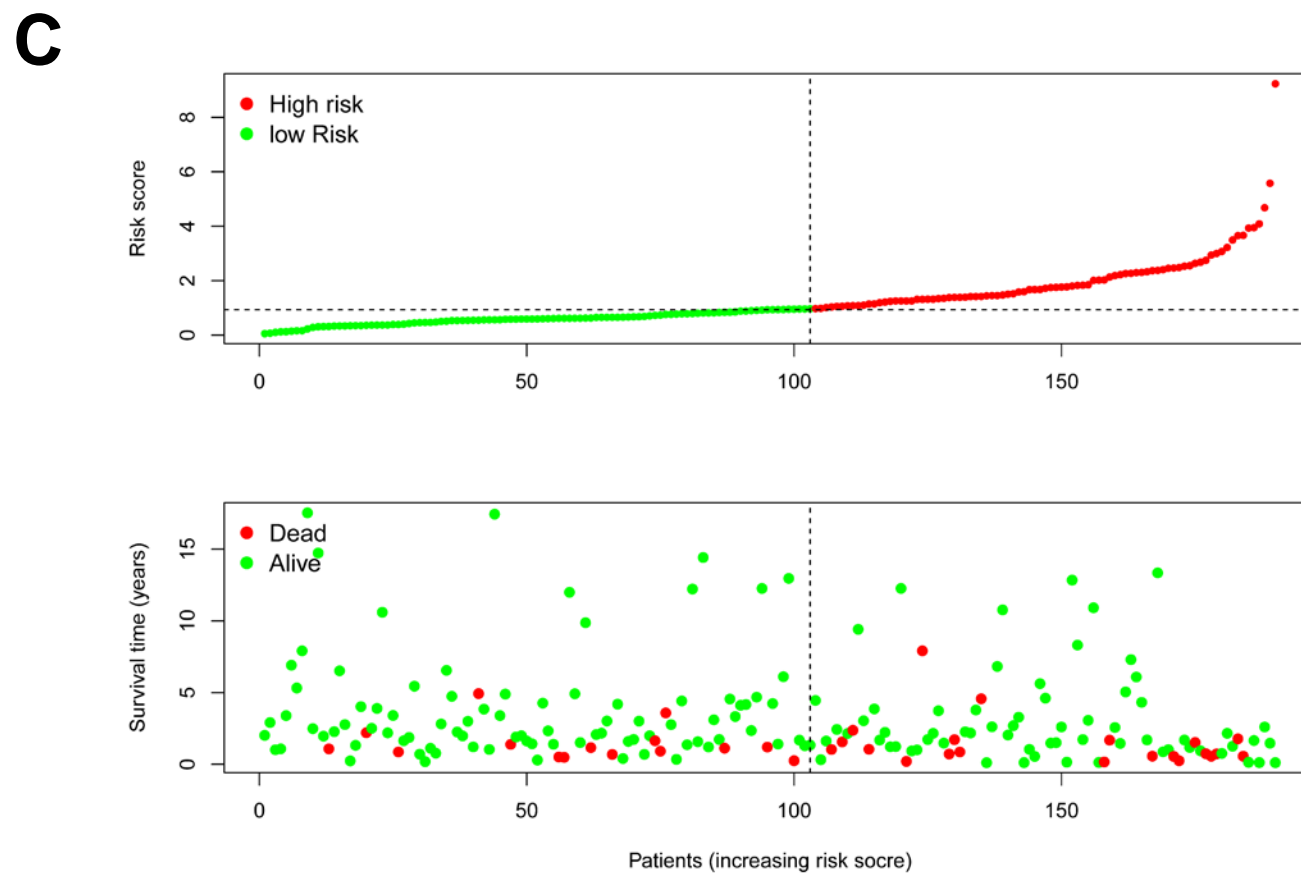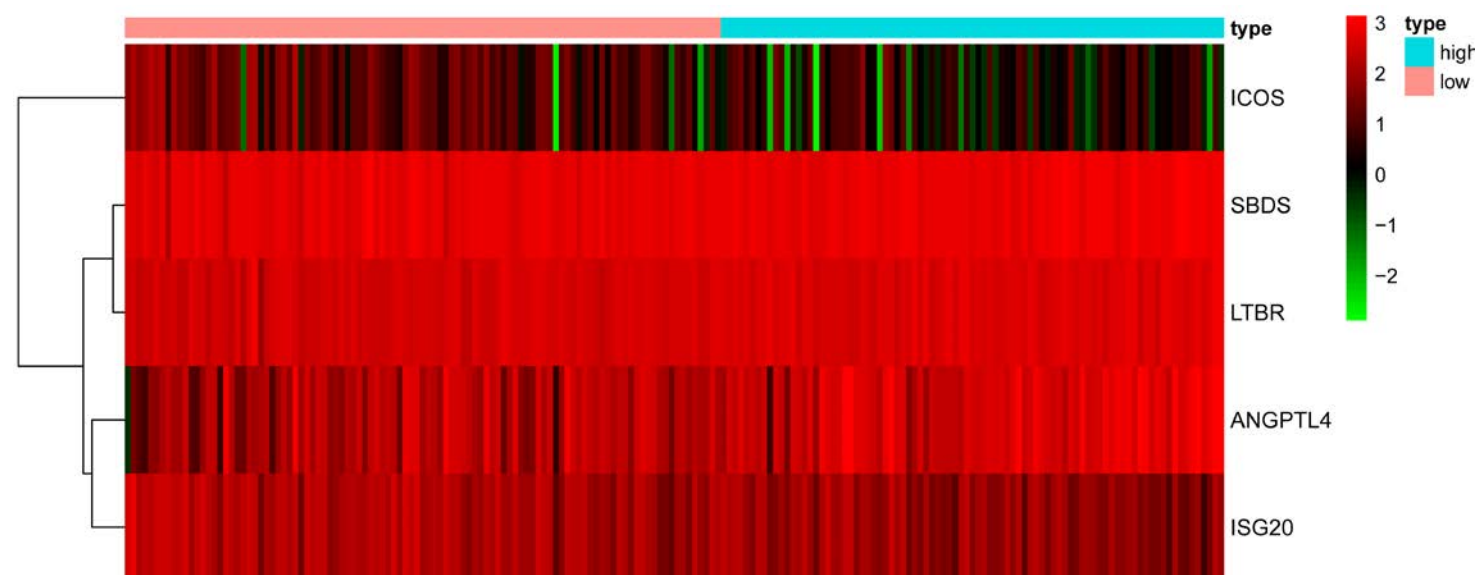

Supplement: Supplementary file 2 [file DataSheet2.PDF]

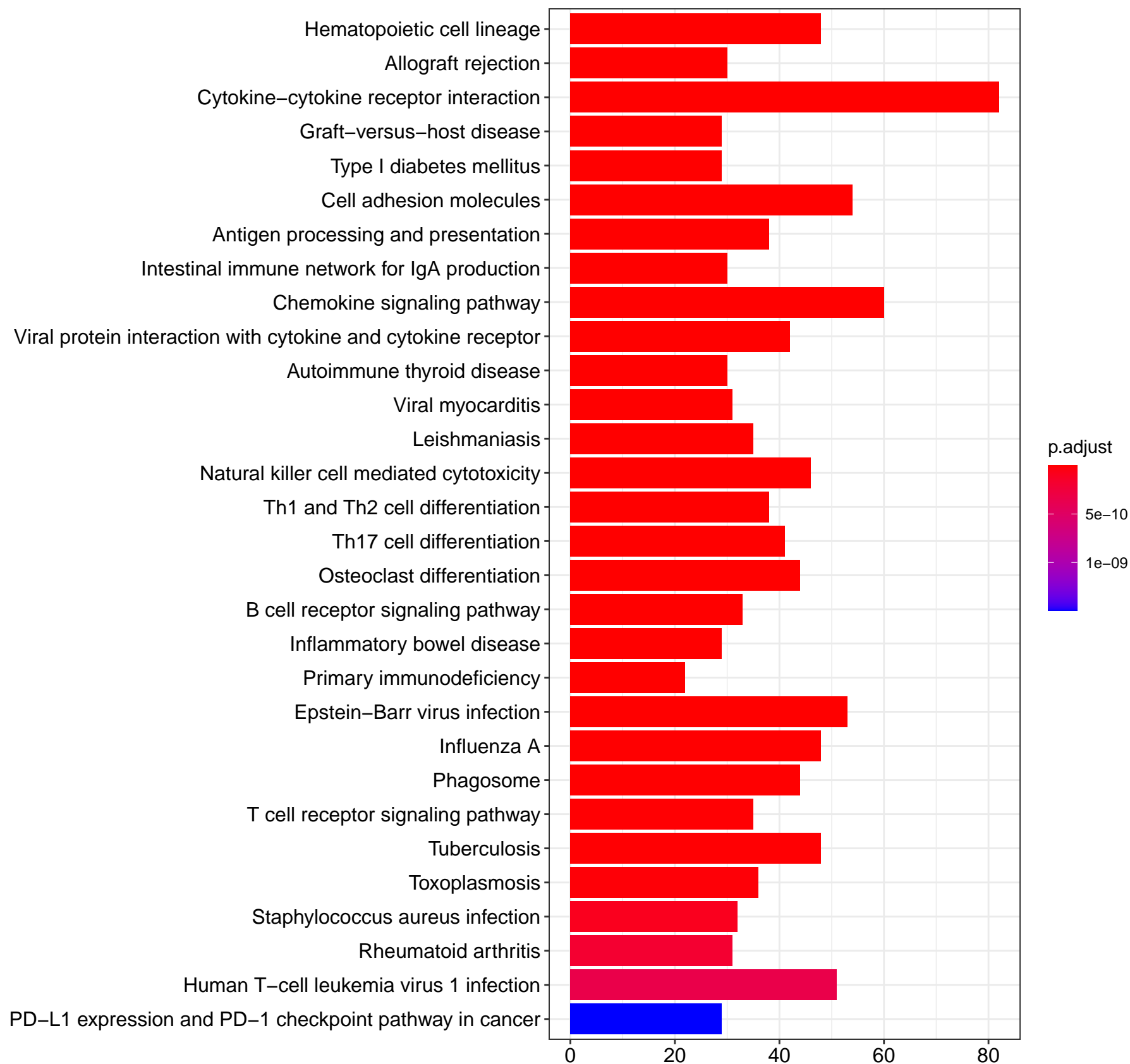

Supplement: Supplementary file 4 [file DataSheet4.PDF]

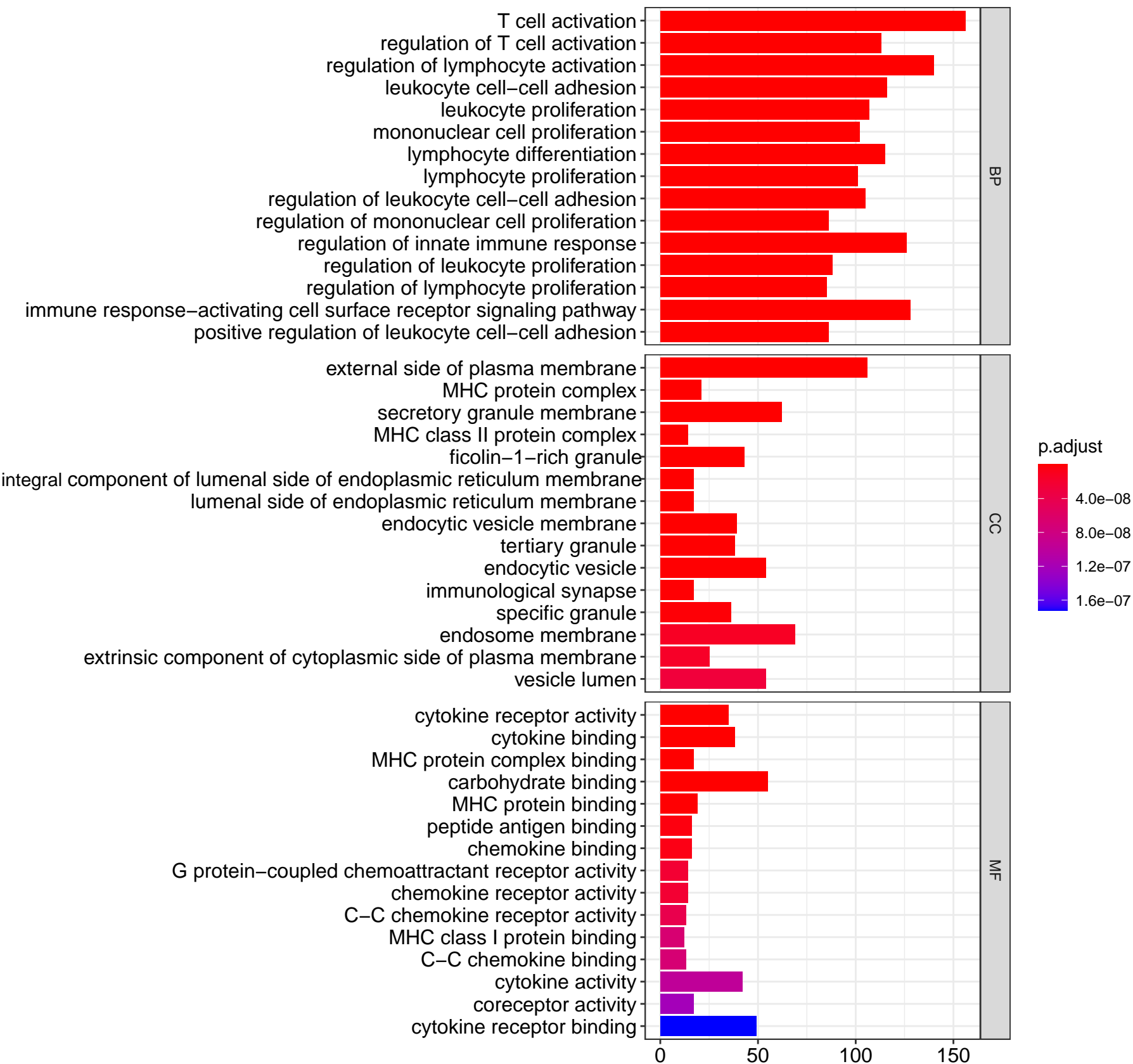

Supplement: Supplementary file 11 [file DataSheet3.PDF]

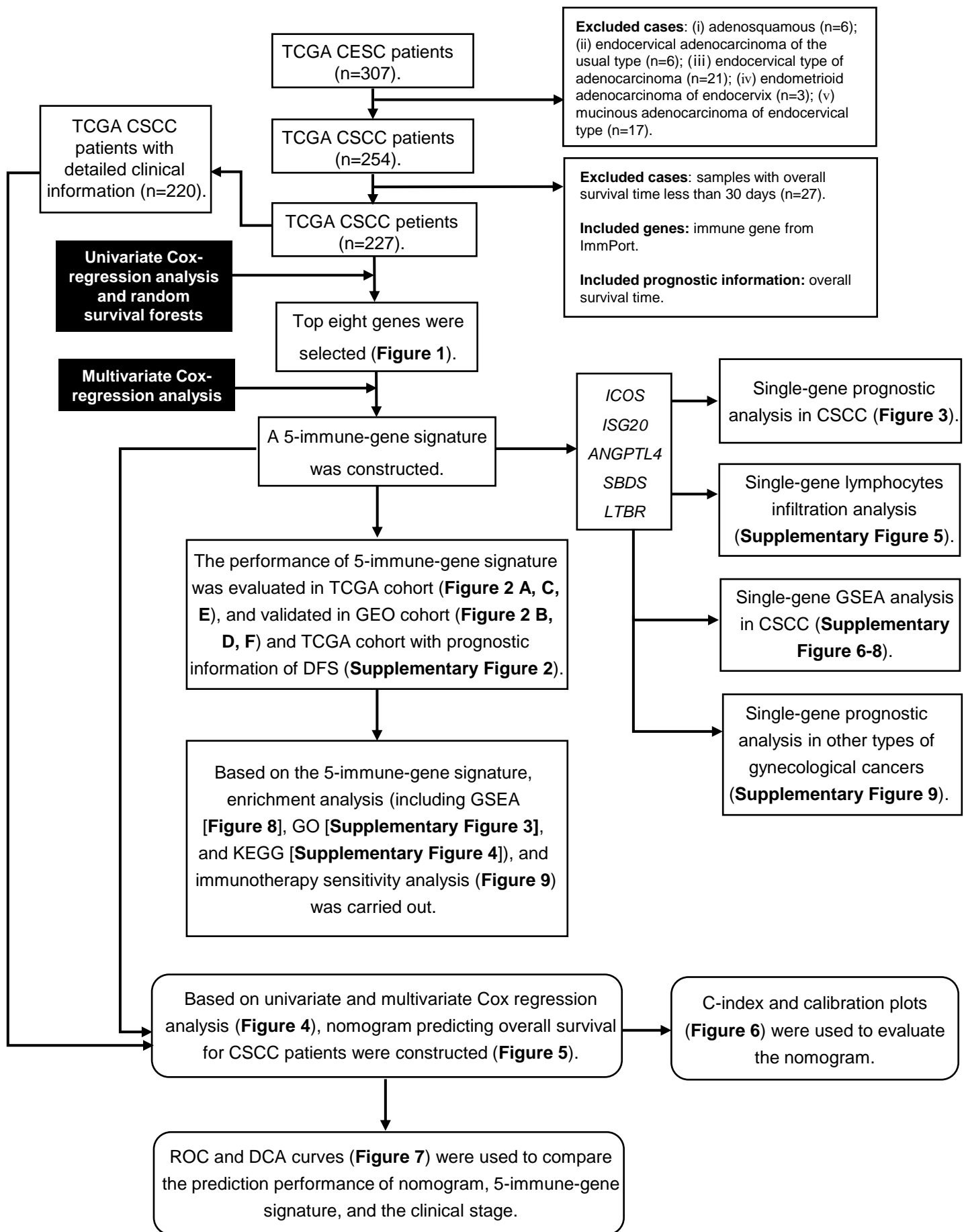

Supplement: Supplementary file 13 [file DataSheet1.PDF]

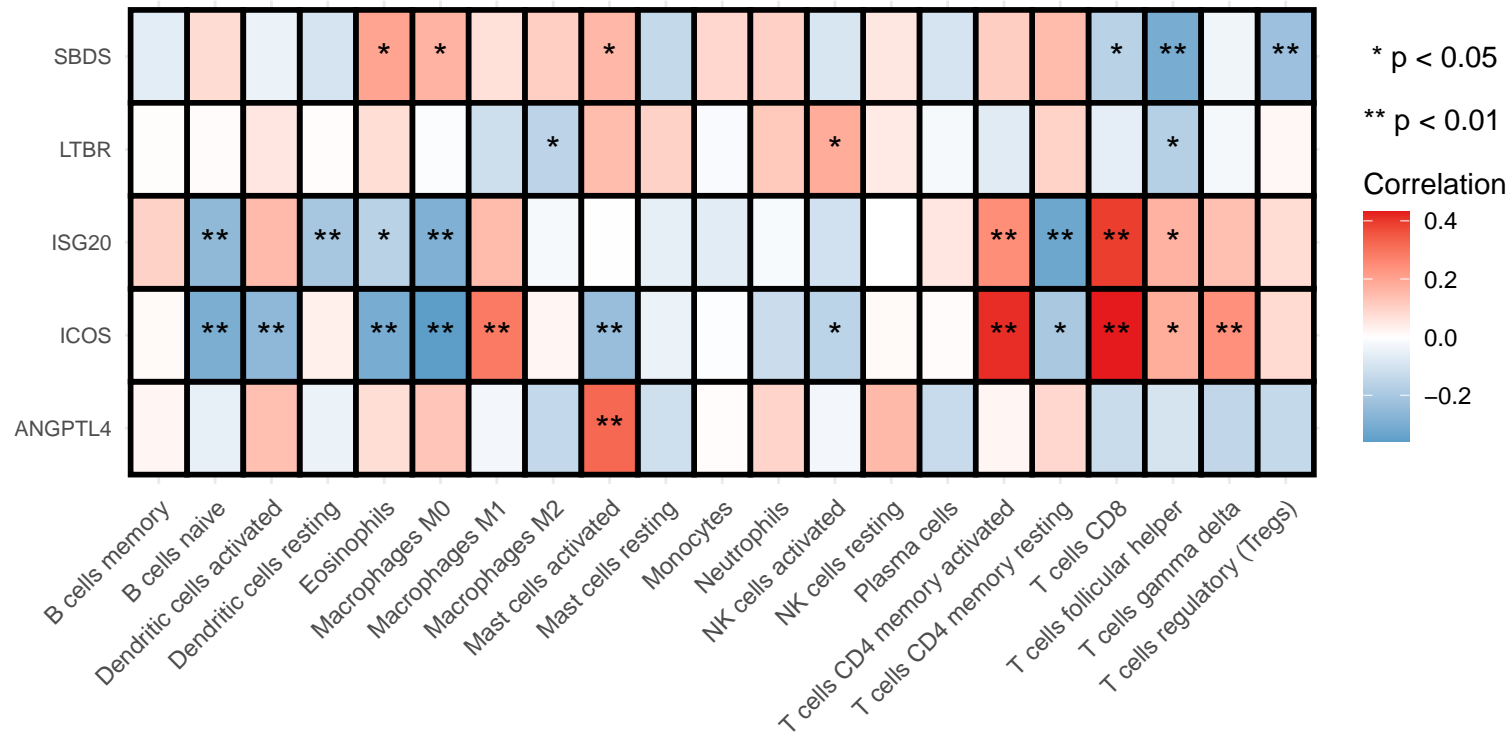

Supplement: Supplementary file 14 [file DataSheet5.PDF]

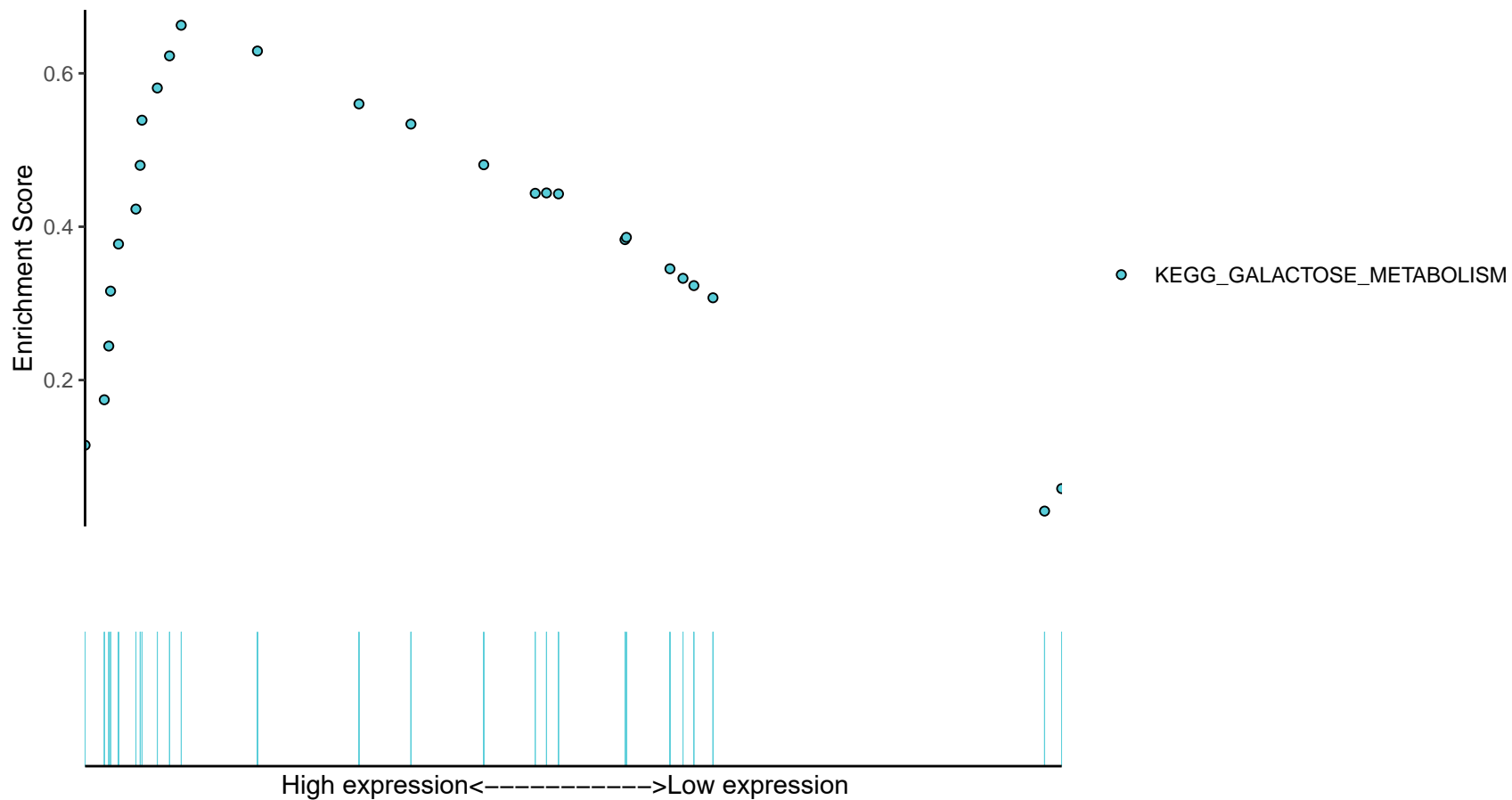

Supplement: Supplementary file 17 [file DataSheet8.PDF]
