# Supplementary material for: An Immune-Gene-Based Classifier Predicts Prognosis in Patients With Cervical Squamous Cell Carcinoma
Source: Front Mol Biosci. 2021 Jul 5;8:679474. doi: 10.3389/fmolb.2021.679474 (PMC8289438; doi:10.3389/fmolb.2021.679474)
Supplement: Supplementary file 5 [file DataSheet6.PDF]

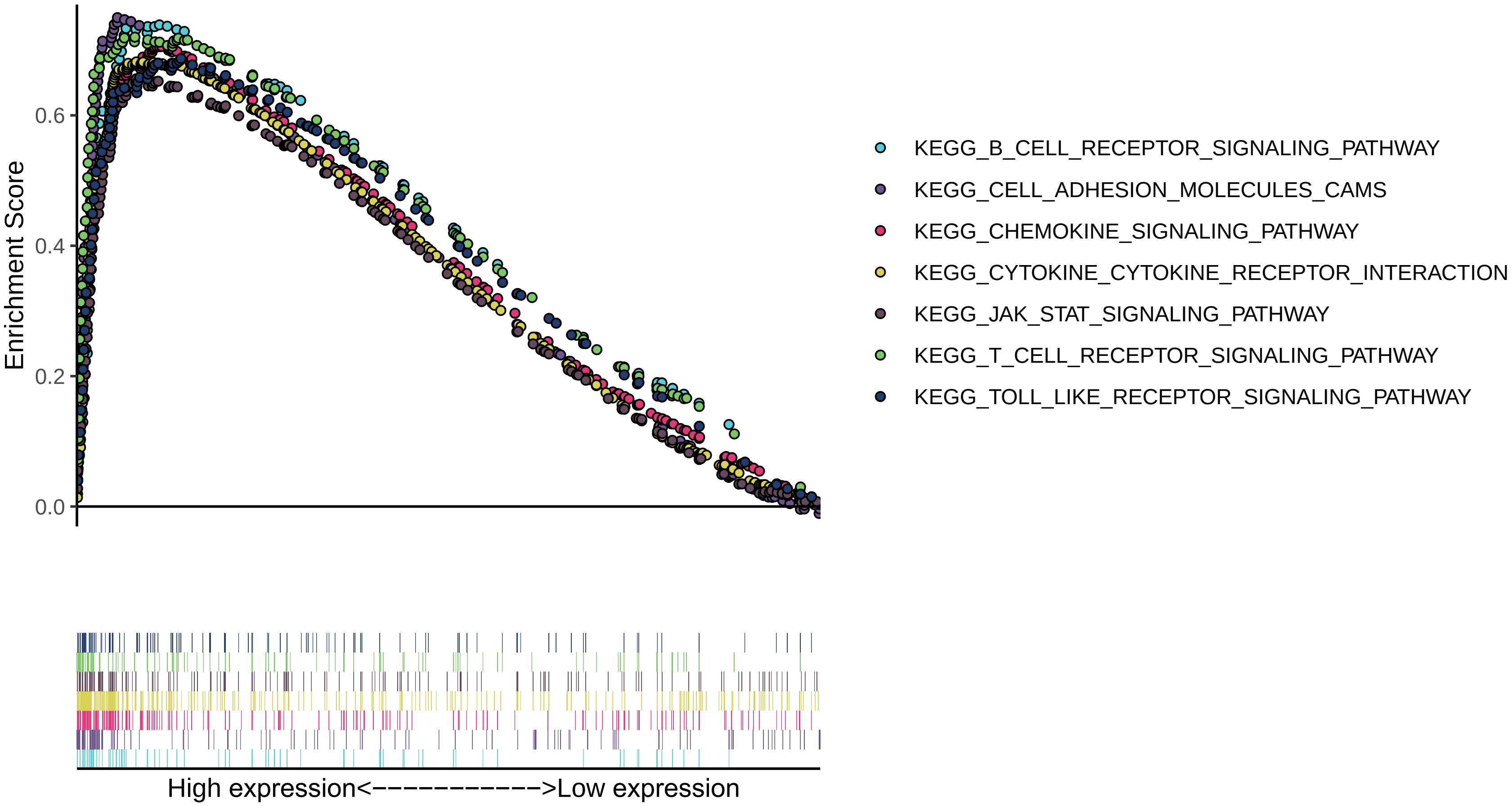

Enrichment Score
0.6
0.4
0.2
0.0
KEGG_B_CELL_RECEPTOR_SIGNALING_PATHWAY
KEGG_CELL_ADHESION_MOLECULES_CAMS
KEGG_CHEMOKINE_SIGNALING_PATHWAY
KEGG_CYTOKINE_CYTOKINE_RECEPTOR_INTERACTION
KEGG_JAK_STAT_SIGNALING_PATHWAY
KEGG_T_CELL_RECEPTOR_SIGNALING_PATHWAY
KEGG_TOLL_LIKE_RECEPTOR_SIGNALING_PATHWAY
High expression<------------>Low expression
