# Supplementary material for: An Immune-Gene-Based Classifier Predicts Prognosis in Patients With Cervical Squamous Cell Carcinoma
Source: Front Mol Biosci. 2021 Jul 5;8:679474. doi: 10.3389/fmolb.2021.679474 (PMC8289438; doi:10.3389/fmolb.2021.679474)
Supplement: Supplementary file 7 [file DataSheet9.PDF]

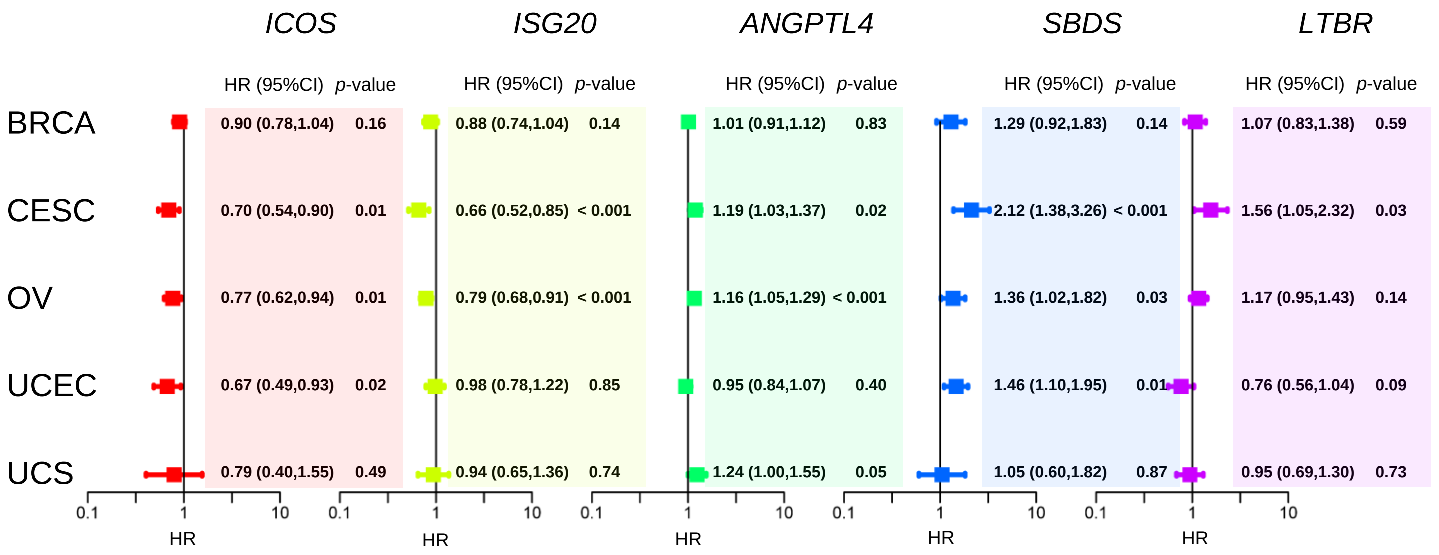
ICOS
ISG20
ANGPTL4
SBDS
LTBR
HR (95%CI) p-value
BRCA
CESC
OV
UCEC
UCS
0.90 (0.78,1.04) 0.16
0.70 (0.54,0.90) 0.01
0.77 (0.62,0.94) 0.01
0.67 (0.49,0.93) 0.02
0.79 (0.40,1.55) 0.49
0.88 (0.74,1.04) 0.14
0.66 (0.52,0.85) < 0.001
0.79 (0.68,0.91) < 0.001
0.98 (0.78,1.22) 0.85
0.94 (0.65,1.36) 0.74
1.01 (0.91,1.12) 0.83
1.19 (1.03,1.37) 0.02
1.16 (1.05,1.29) < 0.001
0.95 (0.84,1.07) 0.40
1.24 (1.00,1.55) 0.05
1.29 (0.92,1.83) 0.14
2.12 (1.38,3.26) < 0.001
1.36 (1.02,1.82) 0.03
1.46 (1.10,1.95) 0.01
1.05 (0.60,1.82) 0.87
1.07 (0.83,1.38) 0.59
1.56 (1.05,2.32) 0.03
1.17 (0.95,1.43) 0.14
0.76 (0.56,1.04) 0.09
0.95 (0.69,1.30) 0.73
0.1
1
10
HR
